# Dietary composition and overlap between cattle and endangered mountain gazelle (*Gazella gazella*)

**DOI:** 10.1038/s41598-025-04366-w

**Published:** 2025-06-06

**Authors:** Amir Arnon, Torsten Wronski, Maya Lalzar, S. Yan Landau, Ido Izhaki, Dan Malkinson

**Affiliations:** 1 Department of Evolution and Environmental Biology, University of Haifa, Haifa, Israel; 2 Ganei Ramat Hanadiv, Zikhron Ya’akov, Zichron Yaakov, P.O. 325, 3095202 Israel; 3 School of Biological and Environmental Sciences, Liverpool John Moores University, Liverpool, UK; 4 Bioinformatics Service Unit, University of Haifa, Haifa, Israel; 5 Department of Natural Resources, Agricultural Research Organization—the Volcani Institute, Rishon leZion, Israel; 6 School of Environmental Studies, and Shamir Research Institute, University of Haifa, Haifa, Israel

**Keywords:** Ruminant diet, Wildlife-livestock interactions, Dietary overlap, Fecal sampling, DNA metabarcoding, Conservation biology, Food webs, Ecology

## Abstract

Israel’s Mediterranean biogeographical region is characterized by high habitat diversity and stark seasonal changes in forage composition, availability and quality. Managers of protected areas in this region advocate livestock ranching to mitigate fire risk and enhance conservation merits. However, competition between livestock and endangered, native ungulates in these areas might impair their functioning as refugia. We used fecal DNA metabarcoding to study the diets of native mountain gazelles (*Gazella gazella*) and domestic cattle (*Bos taurus*), in two nature reserves with distinct vegetation types (shrubland *vs* grassland), and during different seasons. Dietary overlap was ubiquitously low, and seasonal changes in the diets of both ungulates translated into differences in their dietary overlap, with the highest overlap found in grassland during winter. This generally low overlap may be attributed to the extreme differences in their body size or may also result from long-lasting sympatry of gazelles and cattle – first wild and later domesticated—shaping a robust dietary separation. Yet, since cattle biomass is typically much higher than gazelles’, a low dietary overlap in key food items of gazelles may result in their depletion which might negatively affect gazelles, especially during the fawning season and drought years. Our results highlight the need to cover diverse conditions when studying herbivore dietary composition and overlap.

**Supplementary Information:**

The online version contains supplementary material available at 10.1038/s41598-025-04366-w.

## Introduction

Rangelands cover about 45% of the worlds surface, more than any other land use system^1,2^. The combination of supplementary food and water, predator eradication, and veterinary care often maintain artificially high livestock densities, leading to overutilization of resources^3,4^. Because of the extent of livestock ranching and the increased concern for biodiversity, the interactions between livestock and wild herbivores have received increasing attention in recent years^3,5–7^. Livestock might affect sympatric wild ungulates in various, often complex ways, which involve different mechanisms at different spatial and temporal scales. These effects may be direct, e.g., exploitation or interference competition for space, food, and water, or indirect, e.g., modification of habitat quality and productivity, forage quality, and spatial heterogeneity^6,8,9^. Such complexities make it challenging to characterize the relationship between domesticated and wild ungulates that share a common habitat^10,11^. Specifically, dietary composition and overlap between wild and domestic herbivores are difficult to characterize accurately^12^.

The development of novel molecular technologies, namely DNA metabarcoding, enables studies on the dietary composition of various organisms with high resolution, using non-invasive fecal sampling. In recent years, such studies investigated the overlap between the diets of various wild and domestic ungulates in different ecosystems, e.g., African savannas^13,14^, Ethiopian mountains^15^, pine forests in western USA^16^, Mongolian steppe^17^, dry deciduous forests in southern India^18^, or mixed forests in southeastern China^19^. However, while these studies demonstrated a methodological breakthrough and provided valuable insights into the interactions between livestock and wild herbivores, they were mostly conducted in only one habitat type, or during one season. Ungulates, however, constantly modulate their diet composition to balance seasonal changes in their physiological requirements (e.g., growth, mating, pregnancy, lactation) on the one hand^20,21^, and in forage quantity, nutritional values, and palatability on the other^22,23^. Hence, dietary overlap between species is expected to be context-specific, and to differ between seasons^24,25^ and habitats^26,27^. Furthermore, the effects of dietary overlap on individual fitness and population dynamics may also change between habitats and seasons.

Israel’s Mediterranean region encompasses a high habitat diversity with different characteristics (i.e., composition and formation of vegetation and its overall productivity), including semi-arid plains, grasslands, shrublands, woodlands, and planted coniferous forests. The combination of distinct habitats and pronounced seasonality in resource composition, availability, and quality^28–30^ implies a high degree of context-dependent diet composition and overlap between herbivores. Mountain gazelles (*Gazella gazella*) were once common throughout the Levante but have been extirpated by overhunting from most of their historic range and are now classified as ‘Endangered’^31^. Israel is considered home to most remaining mountain gazelle populations, following a successful ban on hunting, and allowing gazelles to persist in several habitats of the Mediterranean region. However, many local gazelle populations experienced a continuous decline and the species’ long-term persistence is jeopardized, due to habitat loss and fragmentation, road kills, and predation by jackals and feral dogs^32^. Moreover, about 50% of protected areas in central and northern Israel (i.e., nature parks and forests), are grazed by livestock, mainly goats and cattle^33^, a practice promoted by land managers to decrease the frequency and intensity of wildfires and their spread into adjacent residencies^34,35^, to restrict bush encroachment^36^, and to increase landscape heterogeneity and biodiversity^37^. The dietary overlap between goats and gazelles was well documented from other Mediterranean areas^38,39^, but that between cattle and gazelles has been ignored since it was assumed that the dietary overlap between a large roughage feeder (cattle) and a selective browser (gazelle) is insignificant. Given the significant differences in body size, physiology, and muzzle width, it is more likely that gazelles selectively browse on plants and forage at different heights than cattle. In contrast, cattle, with their much larger biomass and higher population density, are expected to consume larger volumes of forage, depending on the availability of herbaceous or woody plants at different times of the year or the amount of supplementary feed offered. However, it was shown that cattle-wild browser interactions are complex and variable, ranging from indirect facilitation to direct competition^5,11,40^. Studies from Africa and Asia reported selective browsers—or at least intermediate feeders such as impala^13,41–43^, or sika deer^19^ —to indeed compete with cattle for food resources, including woody plants and perennial herbs. Cattle in Mediterranean woodlands are known to consume large amounts of woody plants^44,45^, suggesting a potential competition for food resources with gazelles. This competition—in conjunction with other factors, namely predation by overabundant predator populations^46^, increased human presence in natural areas^47^, collisions with vehicles, poaching as well as habitat loss and fragmentation^32^—could impede the conservation of gazelles and other wildlife.

In our study, we therefore asked how the diet composition of endangered gazelles and sympatric cattle in two protected areas changed qualitatively between seasons and habitat types, and how these changes translate into dietary overlap between the two herbivore species. Specifically, we predicted that the dietary overlap between gazelles and cattle increases during periods of lowered food availability and/or quality, i.e., in less productive habitats or during the dry season.

## Results

### Diet composition by plant taxa and lifeform

**Fig. 1:**
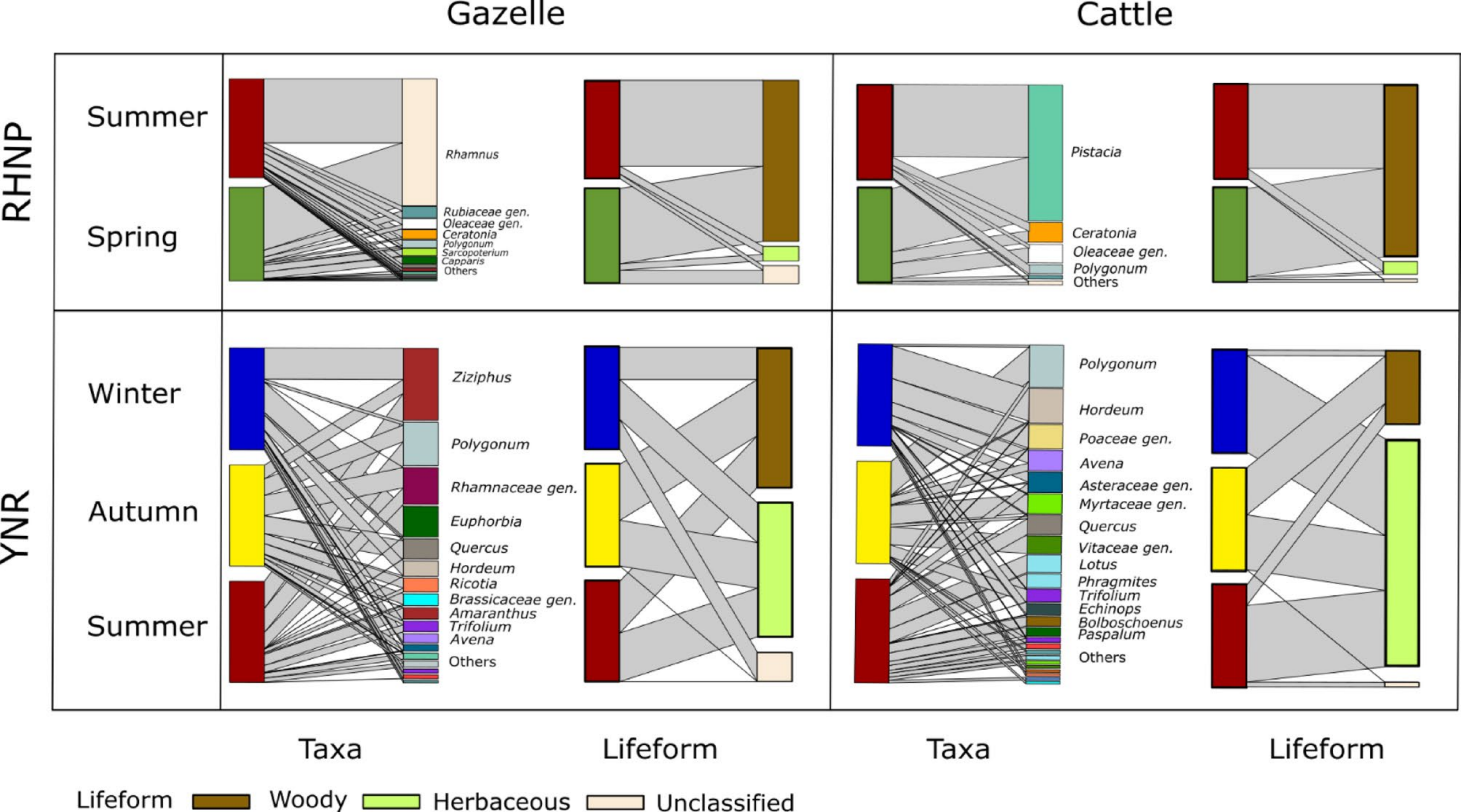
Bipartite networks of diet composition of mountain gazelles (*Gazella gazella*) and cattle (*Bos taurus*) at Ramat Hanadiv Nature Park (RHNP) and Yehudia Nature Reserve (YNR) during the relevant seasons. Networks present plant taxa (at the genus or family level) and lifeforms (herbaceous, woody, or unclassified). Bandwidth represents the average RRA (relative read abundance) of the plant taxon/lifeform in fecal samples. Only taxa for which the mean RRA in at least one season was ≥ 2% were included in both analyses. For gazelles in RHNP, ‘Others’ include *Polygonum*, *Ziziphus*, and *Euphorbia*; for cattle in RHNP, ‘Others’ include *Rhamnus* and Rubiaceae genera.; for gazelles in YNR, ‘Others’ include Asteraceae genera, *Pistacia*, *Capparis*, *Brachypodium*, Poaceae genera, and *Stipa*; for cattle in YNR, ‘Others’ include *Morus*, *Ziziphus*, *Salix*, *Ceratonia*, *Ardisia*, *Trichoneura*, *Ludwigia*, *Pistacia*, and Fabaceae genera.

Seasonal patterns of plant taxa composing the diet of each herbivore (Fig. 1) can be characterized as (i) taxa that were prominent in some of the seasons but not in others, and (ii) taxa with similar proportions in all seasons. Naturally, these compositional changes are also reflected by the proportion of woody and herbaceous plants in the respective diets (Fig. 1).

The gazelle diet in Ramat Hanadiv Nature Park (RHNP) was dominated by *Rhamnus* (especially in spring and summer), by Oleaceae (either *Olea europaea* or *Phillyrea latifolia*) and by Rubiaceae, which were selected in both seasons with similar proportions. *Sarcopoterium* and *Euphorbia* were prominent only in spring, while *Ceratonia*,* Polygonum*, and *Capparis* were only selected during summer. Consequently, woody taxa comprised high proportions of gazelles’ diet in RHNP during both seasons (Fig. 1). The diet of cattle in RHNP comprised almost all woody plant taxa equally in spring and summer. *Pistacia* was the most abundant item in both spring and summer, and the percentage of *Ceratonia* in the diet was also high during both seasons. Oleaceae species were a prominent food item in spring, while *Polygonum* was most prominent in summer. *Rhamnus* was observed in both seasons but with low RRA values.

In Yehudiya Nature Reserve (YNR), woody and herbaceous vegetation constituted similar proportions in the diet of gazelles during all seasons. *Ziziphus* was an important food item in the gazelle diet in all seasons sampled, especially in winter, while other plants of the Rhamnaceae family, as well as *Polygonum* and *Trifolium*, were prominent during summer and autumn but not in winter. The genus *Amaranthus* was prominent in summer, *Quercus* and *Ricotia*, Fagaceae, and Brassicaceae were mainly consumed in autumn, while *Euphorbia*, *Hordeum*, and *Avena* were only eaten in winter. The diet of cattle in YNR constituted higher proportions of herbaceous vegetation than of woody plant taxa, which was primarily prominent in winter. In summer, the proportion of woody taxa increased slightly, and more so in autumn – when the proportion of woody plants almost equaled that of herbaceous taxa (Fig. 1). Members of the Asteraceae were prominent in summer and winter, while *Avena* was chosen in autumn and winter, and *Pistacia* and *Polygonum* in summer and autumn. *Quercus*, *Lotus*, *Phragmites*, *Bolboschoenus*, *Paspalum*, *Cynodon*, *Ludwigia*, *Trichoneura*, *Ziziphus*, and Fabaceae were prominent only in summer. *Trifolium*, Myrtaceae, and Vitaceae were preferred in autumn, while *Hordeum*, *Echinops*, and Poaceae were only consumed in winter.

### Dietary niche partitioning and overlap between gazelles and cattle

**Fig. 2:**
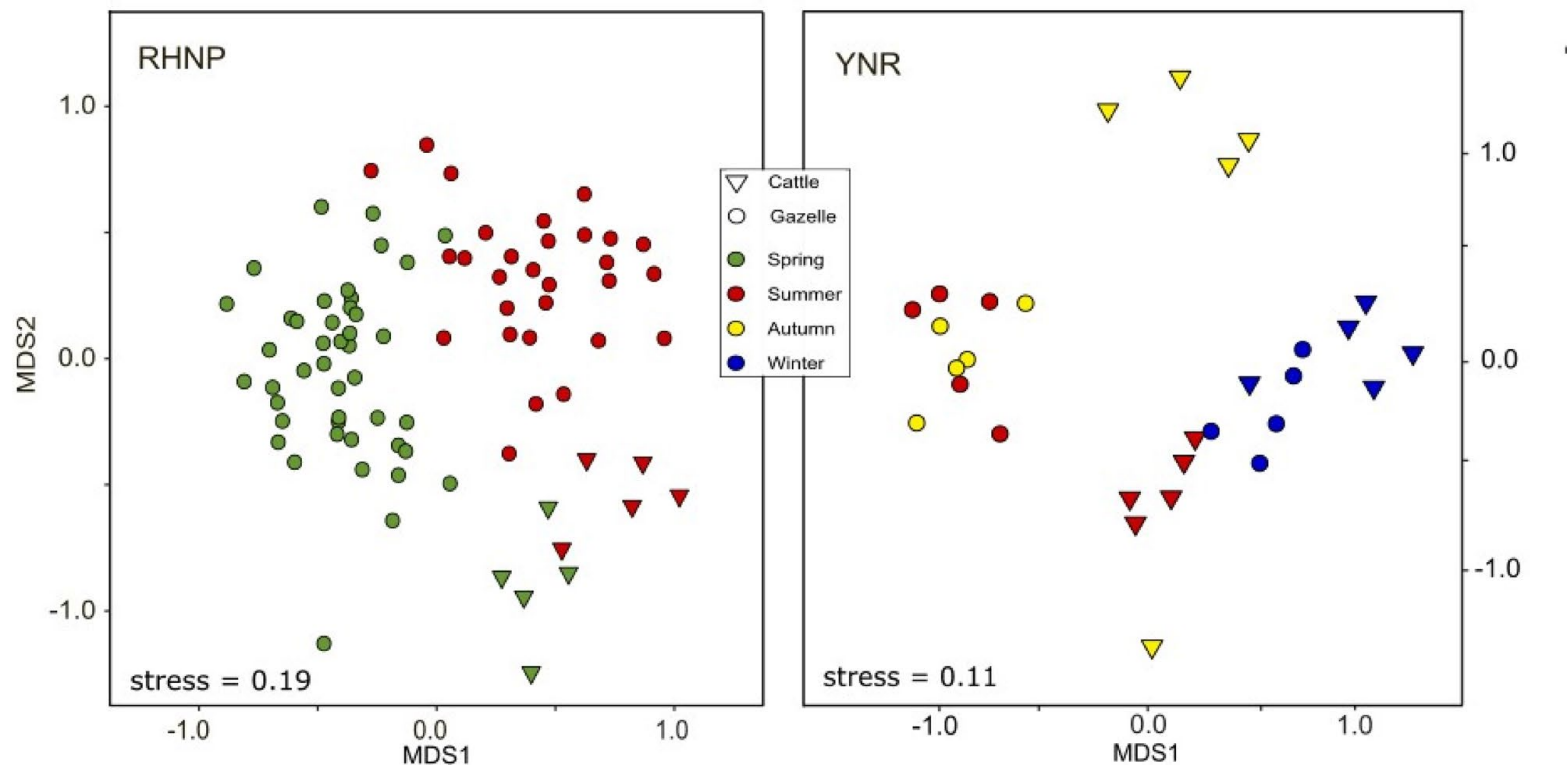
Dietary niche separation between mountain gazelles (*Gazella gazella*) and cattle (*Bos taurus*) in different seasons at two study sites: Ramat Hanadiv Nature Park (RHNP) and Yehudiya Nature Reserve (YNR). NMDS (nonmetric multidimensional scaling) ordination of Bray-Curtis dietary dissimilarity based on Hellinger-transformed readings at the genus or family level. Each point corresponds to one fecal sample.

**Fig. 3:**
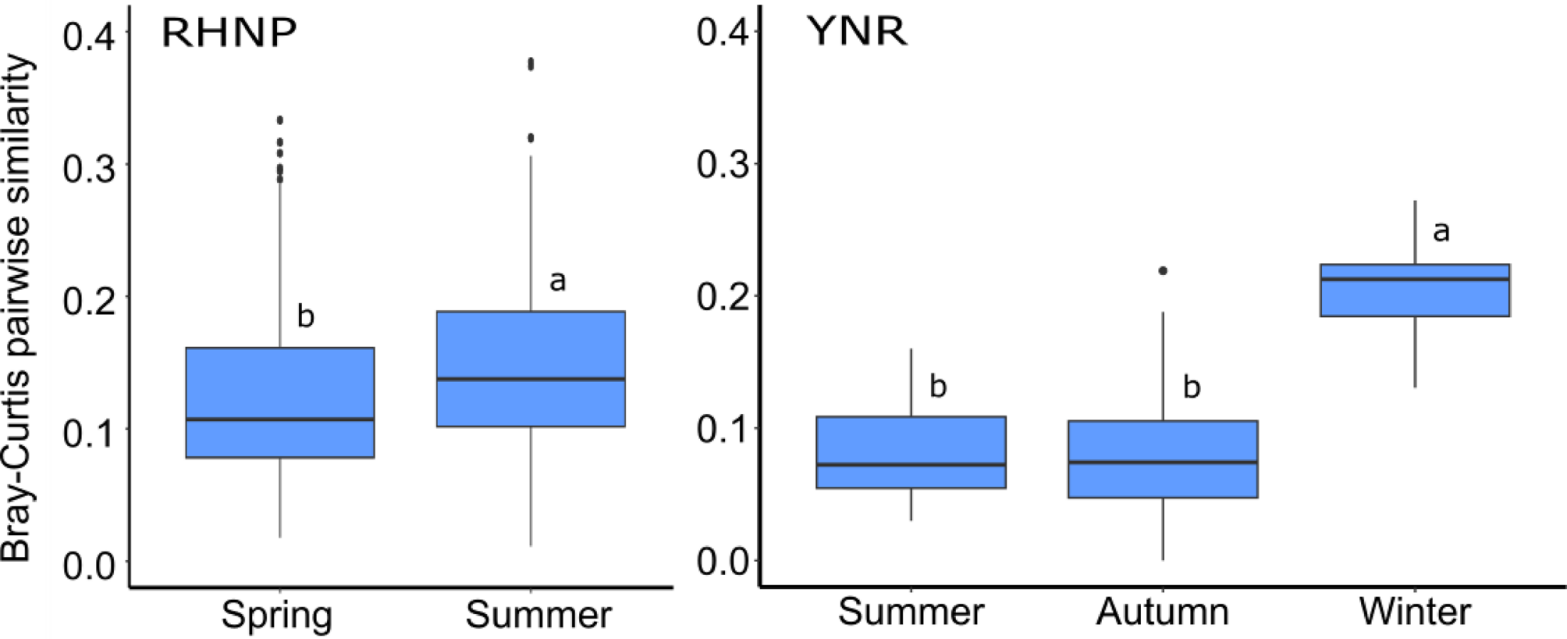
The dietary similarity between mountain gazelles (*Gazella gazella*) and cattle (*Bos taurus*) in seasons when both ungulates co-occur in Ramat Hanadiv Nature Park (RHNP; cattle only present during spring and summer) and in Yehudiya Nature Reserve (YNR; data for spring are missing). Values depicted show Bray-Curtis pairwise similarity (i.e., 1 – Bray-Curtis dissimilarity) between fecal samples, based on relative read abundance (RRA). Within each site, different letters indicate statistically significant differences between seasons (*P* < 0.001), based on the Kruskal-Wallis test and Dunn post hoc test with Bonferroni correction.

Dietary niche partitioning between gazelles and cattle was apparent at both sites (although less so during winter in YNR) as indicated by the large distances between cattle and gazelle samples on both axis (MDS1 and MDS2) of the multidimensional scaling ordination (NMDS; Fig. 2), where distances represent the gradients of variation in dietary composition between samples, i.e., samples clustering together are more similar than those plotted further apart. In RHNP, the clusters of gazelle and cattle were distinct in summer and spring, although the distance between the clusters in spring was smaller on the primary axis (MDS1). The BCsim (Bray-Curtis similarity) indices (Fig. 3) confirmed this trend, i.e., the overlap was significantly higher in summer than in spring, though the difference was negligible (mean BCsim ± SD: 0.15 ± 0.07, and 0.12 ± 0.06, respectively; *P* < 0.001). In YNR similar distances were observed in summer and autumn between the clusters of gazelle and cattle, while the distance between those clusters was relatively small during winter (Fig. 2). The overlap index (Fig. 3) was similarly low in summer and in autumn (mean BCsim ± SD: 0.08 ± 0.04 and 0.08 ± 0.05, respectively; *P* > 0.05) but substantially higher (more than double) in winter (mean BCsim ± SD: 0.21 ± 0.04; *P* < 0.001).

The dietary niche partitioning between gazelles and cattle in the two ecosystems was further confirmed by the PERMANOVA (Table 1). In both, RHNP and YNR, season, ungulate species, and the interaction term of both were significant (*P* < 0.01). The NMDS plots, and the results of PERMANOVA and Kruskal-Wallis tests, based on the plant taxa’s presence or absence (Jaccard’s index), were similar to those obtained for both sites (Figure S1, Figure S2, Table S1).

**Table 1:** Permutational analyses of variance for dietary differnces between ungulate species (gazelles and cattle), season, and for the species × season interaction term. Analyses were based on Bray-Curtis dissimilarity distances of Hellinger-transformed readings, calculated for seasons in which the two ungulate species co-occurred, i.e., spring and summer in Ramat Hanadiv Nature Reserve (RHNP), autumn, spring and winter in Yehudiya Nature Reserve (YNR). P: ‘*’ < 0.05, ‘**’ < 0.01, ‘***’ < 0.001.

RHNP				YNR				
Factor	df	sums of sqs	R^2^	Factor	df	sums of sqs	R^2^	
Species ***	1	1.94	0.07	Species ***	1	1.26	0.11	
Season ***	1	3.06	0.11	Season ***	2	2.43	0.22	
Species × season **	1	0.64	0.02	Species × season ***	2	1.61	0.14	
Residuals	79	21.49	0.80	Residuals	24	5.86	0.53	
Total	82	27.14	1.00	Total	29	11.16	1.00	

## Discussion

**Fig. 4:**
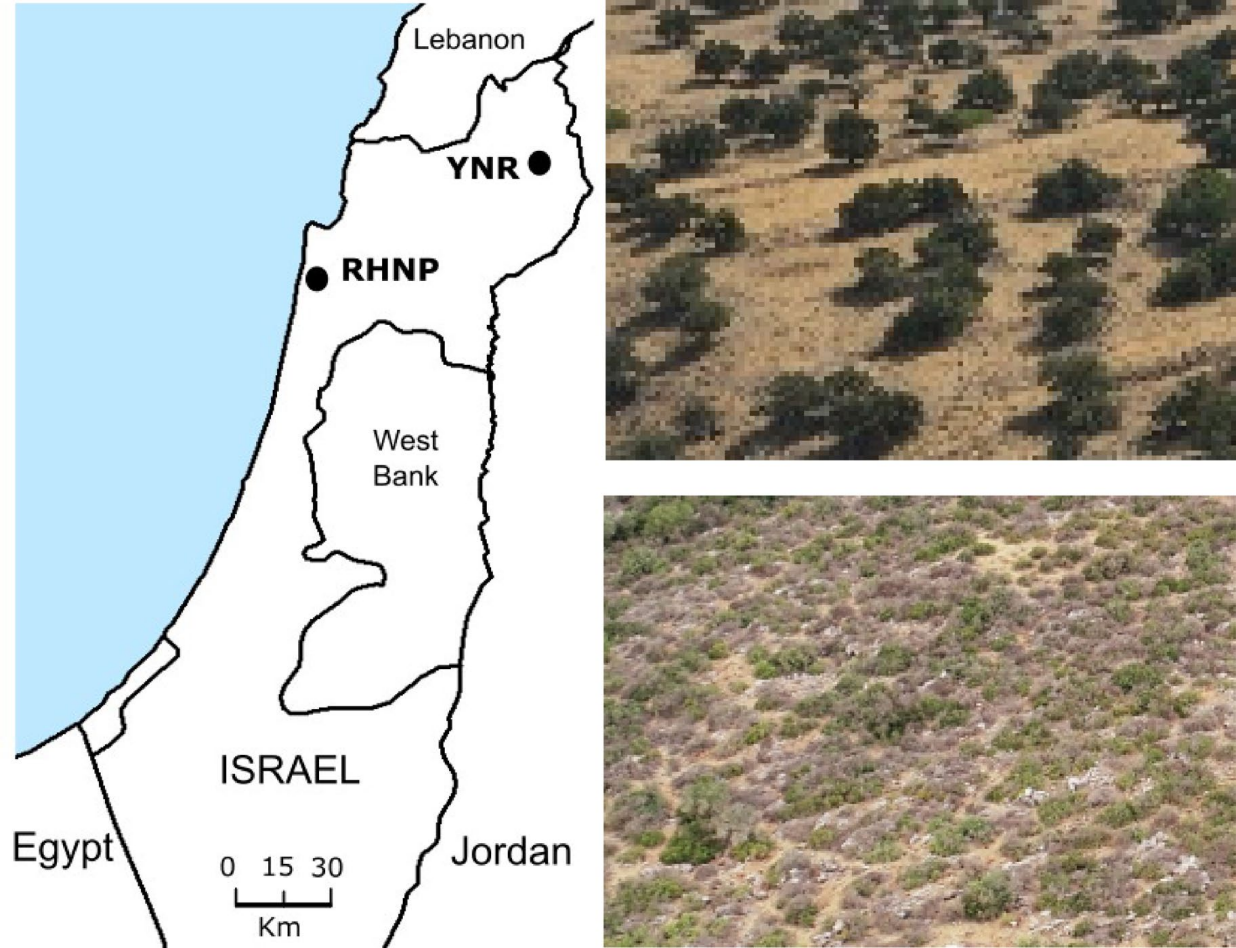
The locations of the study sites in northern Israel and aerial photos of their landscape characteristics: open park/grassland interspersed with oaks at Yehudiya Nature Reserve (YNR; top), and garrigue at Ramat Hanadiv Nature Park (RHNP; bottom).

Although the interpretation of our results must be considered with caution due to small sample sizes, one noticeable pattern is the pronounced dominance of one plant taxon during spring and summer in RHNP, namely *Rhamnus* for gazelles and *Pistacia* for cattle (Fig. 1). In YNR, we unrevealed a dominance of *Ziziphus* and *Euphorbia* in the winter diet of gazelles, of *Hordeum* and other Poaceae in the winter diet of cattle, and of *Polygonum* in the autumn diet of cattle, however, a dominance much less prominent than that observed in RHNP. This pattern can be attributed to the different vegetation prevailing in either ecosystem, whereby the availability and nutritional attributes of most woody plants in the Mediterranean region remain generally stable throughout the year^33,48^. Herbaceous plants, on the other hand, are abundant in late winter and spring, a time when they are highly palatable and digestible, and when they have high water, protein, energy, and mineral contents, while during summer and autumn their biomass and nutritional quality decrease dramatically^29,33^. Since woody vegetation is dominant in the garrigue of RHNP (see Fig. 4), ruminants can base their diet on one or a few woody genera with stable quality and availability^49^. In contrast, in the grass-dominated landscape of YNR (Fig. 4), they need to consume more herbaceous plants when those are available and nutritious, i.e., in winter and spring^30^. These patterns are consistent with former studies^50^, which showed that gazelles in RHNP consumed similar proportions of woody and herbaceous plants across seasons, while in the northern grassland, gazelles consumed mostly annuals in winter, spring and early summer, but from mid-summer to early winter they preferred perennial herbs, shrubs, and trees^51,52^. The dietary responses to seasonal changes in the availability of forage seem to differ between gazelles and cattle, as indicated by the significant ungulate × season interaction term revealed by the PERMANOVA tests (Table 1) and by the clustering illustrated in the NMDS plots. Figure 2, for example, shows that the clusters for gazelles of RHNP in spring and summer are much further apart than those of cattle, which reflects a more noticeable change in the dietary composition of gazelles than in that of cattle. However, summer preferences may be biased by the fact that poultry litter is given, prompting cattle to consume dry, withered, nitrogen-poor forage.

Differences in the dietary composition of gazelles and cattle may be attributed to the extreme differences in their body size. Various allometric relationships between body mass, metabolic requirements, and digestive efficiency imply that larger ruminants can subsist on food of lower quality (roughage feeder), while small ones are more selective, utilizing more digestible forage, which is one of the main mechanism enabling the coexistence of sympatric ruminants^53–57^. Yet, several metabarcoding studies on the dietary overlap between cattle and indigenous herbivores found much higher overlaps than would be expected by their body mass. For example, a Pianka’s index^58^ of 63% was reported between cattle and elk (*Cervus canadensis*) and of 51% between cattle and mule deer (*Odocoileus hemionus*)^16^. Moreover, 84% overlap by Pianka’s index and 63% by BCsim was reported between cattle and sika deer (*Cervus nippon*)^19^, and 68% overlap by BCsim was found between cattle and sambar deer (*Rusa unicolor*)^18^. Although we did not use Pianka’s index for dietary niche overlap—it compares pools of plant taxa consumed by either ungulate and is thus sensitive to small sample sizes—the high overlap reported by studies using Pianka’s index is striking, compared to the values we observed.

In addition to size differences, another possible explanation for the low dietary niche overlap might be the realization of past processes (e.g., the ghost of competition past^59^, which on an evolutionary timescale led to decreased dietary overlap between two long-term coexisting species^25,60^. Mountain gazelles shared their ranges throughout the Levante for millions of years, first with wild cattle (aurochs; *Bos primigenius*—extinct from the Levante around 3200 to 2600 BP^61^, and later with its descendant, the domestic cattle, which roams the Middle East for millennia^62^, i.e., since its domestication in the Fertile Crescent around 10,000 years BP^63^. The dramatic increase in dietary overlap in YNR from 8% in summer and autumn to 21% in winter (i.e., the rainy season in Mediterranean ecosystems) is consistent with this scenario, since with higher resource availability, dietary overlap between species with long-term coexistence is expected to increase^64^.

As cattle grazing is practiced in many protected areas of Israel, a high dietary overlap with gazelles raises concerns regarding the persistence and reproduction of endangered gazelles in areas designated for conservation. In our study we documented low levels of dietary overlap between cattle and gazelles, which implies they do not compete for food. The fact that we found low overlap values in very different contexts—two ecosystems that differ in vegetation structure and productivity, and during different seasons—confirms the general validity of our conclusions. However, the interpretation of this finding should be viewed with caution. The daily food consumption of an adult mountain gazelle is roughly 440 g of dry matter per day (averaging males and females)^65^, while that of cattle is 9.25 kg per day^44^, i.e., about 21 times higher than in gazelles. Moreover, as the total biomass of cattle in the ecosystem could outweigh that of gazelles by orders of magnitude—cattle roughly have a 20 times higher individual weight and occur typically in much higher densities than gazelles—even a low dietary overlap could have profound effects on the nutrition of gazelles. Some plant taxa are essential for gazelles, and the indiscriminate consumption of forage by cattle could translate into a depletion of key food items, especially when these items are scarce.

Though we could not directly compare the two study sites, because the occurrence of herbivores—depending on the cattle herding regime—differed between seasons, our study highlights the benefits of examining the overlap between wildlife and livestock under diverse conditions. However, a major shortcoming of our study was the low sample size, which was (except for gazelle samples from RHNP) due to methodological and administrative constraints (see below). Despite the statistically highly significant differences detected by our analyses, which imply that our findings were not coincidental, caution should be taken when interpreting these results. Future research should thus strive to include more comparable conditions between study sites and seasons and could capitalize on the possibility to determine the sex or even the identity of individuals from fecal samples, which is still technically challenging. Such data would enable high-resolution research on aspects such as sexual, or other intra-species differences. Finally, given that our study was conducted under relatively mild climatic conditions, i.e., rainfall was between 100 and 130% of the average^66^, it might well be that under harsher conditions the degree of dietary overlap would have been more prominent.

## Materials and methods

### Study species

Mountain gazelles (*Gazella gazella*) are small-sized ungulates of the Bovidae family (mean weight ± SD: 24.9 ± 3.6, and 18.1 ± 2.6 kg for adult males and females, respectively^67^). Gazelle diet comprises a wide variety of plants, reflecting the flora at specific habitats and seasons. In the garrigue of central Israel, Geffen^50^ reported gazelles to feed on several woody genera, such as *Pistacia*,* Ceratonia*,* Rhamnus*,* Calicotome*,* Ziziphus*, and *Phillylea*, as well as various herbaceous plants, while in the semi-arid habitat of Lower Galilee, gazelles feed mainly on grasses and forbs, but also on *Zizphus*,* Prosopis*, and *Alhagi*^51^. Gazelles use scent-mark stations (localized defecation sites) for social communication and to demarcate male territories^50,68,69^. Free-ranging cattle (*Bos taurus*) in Israel have an average weight of approximately 550 kg^70^, feeding year-round on natural vegetation but are typically provided with supplementary feed when the herbaceous vegetation withers at the end of the summer^29^.

### Study sites

Our study encompassed two protected areas in Israel, about 85 Km apart (Fig. 4): (1) Ramat Hanadiv Nature Park (RHNP; 32°30′N; 34°57′E) comprising about 480 ha, located in the coastal area on a rocky plateau at a mean elevation of 120 m a.s.l and with no or very shallow soils. The climate is east-Mediterranean, with an average annual rainfall of 550 mm, mainly between November and March. The primary vegetation formation is shrubland (garrigue), dominated by low evergreen trees such as *Phillyrea media*, *Pistacia lentiscus*, and *Rhamnus lycioides*. Other species include low-growing dwarf shrubs (e.g., *Calycotome villosa* and *Sarcopoterium spinosum*), and a high diversity of short-lived annuals, perennial grasses and geophytes^71^. About 200 goats roam the area for approximately four hours a day, therefore representing no—or little—competition for the gazelles, although their spectrum of food plants potentially overlaps^38,39^. Seasonal cattle grazing was introduced to RHNP in 1989 following a major wildfire in the early 1980 s^72^. The herd, of about 200 individuals, typically roams the park between late winter (February) and early summer (June-July). The average cattle stocking rate in the park during the study period was 84.2 cow grazing days per hectare. Gazelle densities at RHNP were estimated at around 10 individuals per hectare (Arnon, A., unpublished data). (2) The Yehudiya Nature Reserve (YNR; 32°56′N; 34°27′E), is a 6,600 ha protected area in the Golan Heights, approximately 150 m a.s.l., with a mean annual precipitation of 540 mm and very dry but fertile soils (luvisol-xersol associations). The primary vegetation is a savanna-like woodland, dominated by woody evergreen *Quercus ithaburensis*, *Ziziphus spinachristi*, and *Z. lotus*, interspersed by a matrix of herbaceous species dominated by *Avena sterilis*, *Hordeum bulbosum*, and *Trifolium pilulare*^73^. Goats were absent from the area, while cattle grazing was year-round, with an average stocking rate of around 152 cow grazing days per hectare. Gazelle densities at YNR were estimated at around 14 individuals per hectare (Goldstein, H. personal comm.).

### Sample collection and preparation

We collected fecal samples of gazelles and cattle along 4 to 6 km transects walked throughout each study site for several days during each sampling season in 2019–2020 (Table 2). Due to regulations of the Nature and Parks Authority, sample collection in YNR was only possible along hiking trails. We collected 10 to 30 pellets from gazelle mounds and about 30 g fecal material from each cattle pat. Samples were put in paper bags and transferred to a freezer at −20˚C within six hours until further processing. To ensure the representation of different individuals, we chose sampling locations spatially scattered across each study site (Figure S 3). For gazelles, samples were collected from inside and outside localized defecation sites to ensure both sexes were equally represented. All samples chosen for analyses were dried at 50 °C for 48 h. To meet USDA regulations for importing ungulate feces, samples were further dried at 72 °C for 30 min before being sent to Jonah Ventures lab, Boulder Colorado, USA; USDA import permits 138410, 142960). To facilitate the identification of local plant species in fecal samples, reference samples of 53 Mediterranean plants known as important dietary components of mountain gazelles^50,51^ and cattle^74^ were collected in RHNP, sent to the lab for metabarcoding, and included in the Jonah Ventures voucher sequence records.

**Table 2:** Number of fecal samples processed and analyzed in this study (collected from 2019 to 2020), presented for each ungulate species (gazelles and cattle), season, and study site. Season were defined as spring (May-June), summer (July-August), autumn (September-October), and winter (January-February).

		Spring	Summer	Autumn	Winter
RHNP	Gazelle	48	31	-	-
	Cattle	5	5	-	-
YNR	Gazelle	-	5	5	5
	Cattle	-	5	5	5

### Metabarcoding

Genomic DNA was extracted from samples using the DNeasy PowerSoil HTP 96 Kit (Cat # 12955-4) according to the manufacturer’s protocol and eluted into 100µl and frozen at −20 °C. A portion of the chloroplast trnL intron from each genomic DNA sample was PCR amplified using the c and h trnL primers. Both forward (CGAAATCGGTAGACGCTACG) and reverse (CCATTGAGTCTCTGCACCTATC) primers^75^ also contained a 5’ adaptor sequence to allow for subsequent indexing and Illumina sequencing. Each 25 µL PCR reaction was mixed according to the Promega PCR Master Mix specifications (Promega catalog # M5133, Madison, WI), which included 0.4 µM of each primer and 1 µl of gDNA. DNA was PCR amplified using the following conditions: initial denaturation at 94 °C for 3 min, followed by 40 cycles of 30 s at 94 °C, 30 s at 55 °C, and 1 min at 72 °C, and a final elongation at 72° C for 10 min. Each reaction was visually inspected to determine amplicon size and PCR efficiency using a 2% agarose gel with 5 µl of each sample as input. Amplicons were then cleaned by incubating amplicons with Exo1/SAP for 30 min at 37 °C, followed by inactivation at 95 °C for 5 min, and stored at −20 °C.

A second round of PCR was performed to give each sample a unique 12-nucleotide index sequence. This PCR included Promega Master mix, 0.5 µM of each primer, and 2 µl of template DNA (cleaned amplicon from the first PCR reaction). It consisted of an initial denaturation of 95 °C for 3 min followed by eight cycles of 95 °C for 30 s, 55 °C for 30 s and 72 °C for 30 s. Final indexed amplicons from each sample were cleaned and normalized after visual inspection of 5 µl of indexing PCR product on a 2% agarose gel, using SequalPrep Normalization Plates (Life Technologies, Carlsbad, CA). 25 µl of PCR amplicon were purified and normalized using the Life Technologies SequalPrep Normalization kit (cat#A10510-01) according to manufacturer protocol. Subsequently, samples were pooled by adding 5 µl of each normalized sample.

Sample library pools were sent for sequencing on an Illumina MiSeq (San Diego, CA) in the CU Boulder BioFrontiers Sequencing Center using the v2 300-cycle kit (cat# MS-102–2002). Necessary quality control measures, which include AmpPure bead cleanup to remove non-specific < 200 bp amplicons, Qubit quantitation, and Tapestation amplicon average size analysis, were performed at the sequencing center before sequencing. For sequence raw data processing, sequencing success and read quality were verified using FastQC v0.11.8 and reads were demultiplexed by Illumina-utils v2.6 (iu-demultiplex) using default settings. Sequences of each sample were then merged using the -fastq_mergepairs option in Usearch v11.0.667^78^. The forward primer (5’- CGAAATCGGTAGACGCTACG-3’) and reverse primer (5’- CCATTGAGTCTCTGCACCTATC-3’) were removed using Cutadapt v1.18^79^, which was also used to discard sequences with lengths below 108 bp. Usearch was then used to discard low-quality reads (max_ee = 0.5^78^;, and reads affected by sequencing and PCR errors were removed using the unoise3 algorithm with an alpha value of 5^81^. This denoising was applied to each sample, and Exact sequence variants (ESV) were compiled in an ESV table, including sequences and read counts for each sample. Taxonomy was assigned to each ESV by mapping them against GenBank reference data^80^ as well as Jonah Ventures voucher sequences records, using usearch_global with –maxaccepts 0 and –maxrejects 0 to ensure mapping accuracy. Consensus taxonomy was generated from the hit tables by first considering 100% matches and then going down in 1% steps until hits are present for each ESV. In the respective 1% bracket, taxonomy present in at least 90% of the hits was reported, or an NA, if several taxa match the ESV. To reduce errors caused by misidentified taxa, the bracket was increased to 2% if matches of 97% or higher were found, and no family-level taxonomy was returned.

### Sample freshness and metabarcoding performance

**Fig. 5:**
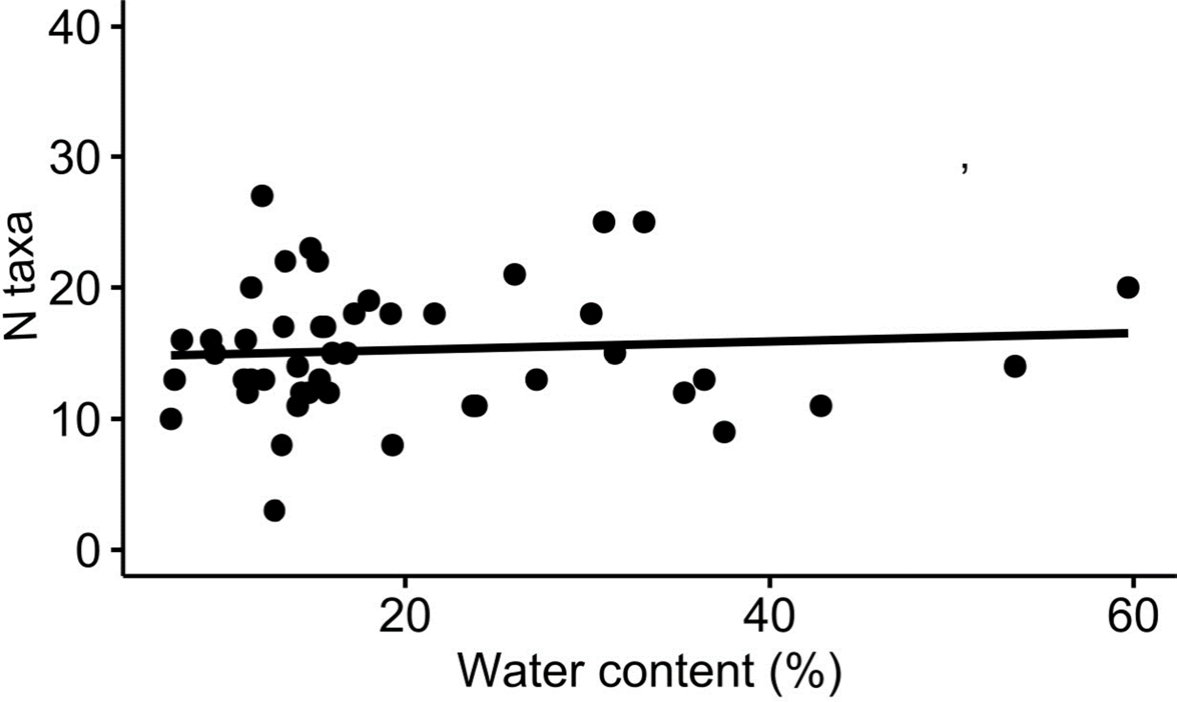
The water content of 45 mountain gazelles (*Gazella gazella*) fecal samples and the number of recovered plant taxa. Samples were collected in the spring and summer of 2019 in Ramat Hanadiv Nature Park.

In Metabarcoding studies, emphasis is often given to collecting fresh samples^14,17,19^. Mountain gazelles, however, are sparsely distributed throughout the landscape and often reside in thick vegetation, which encumbers obtaining fresh samples, especially outside latrines^81^. To confirm that sample freshness did not affect our results, we calculated the water content of 45 gazelle fecal samples from the spring and summer of 2019, by weighing them prior and after drying, and used it as a proxy for sample freshness. We found no significant relationship (Pearson’s *r* = 0.13, *P* = 0.38; Fig. 5) between a samples’ water content (*N* = 45, mean = 20.07% ± 11.76 SD, range = 7.1–59.7%) and the number of plant taxa, at genus or family level, which validates the use of samples of varying freshness, from very fresh (wet) to quite old (dry).

### Data analyses

The identification of plants to the species level using the trnL marker is often ambiguous, especially for several plant families that are important for the study species (namely Poaceae, Asteraceae, and Fabaceae^14,75,82^;. Therefore, all analyses were done at the genus level. When a sequence could not be assigned a genus (very few sequences), we included the family or order as a taxon. In total, 1548092 high quality reads were obtained and binned into 1183 ESVs. Of those, 858 ESVs, representing 97.5% of the total reads (1509602 reads), were taxonomically assigned to at least the order level. Therefore loss of data was minimal. Accordingly, 170 taxonomic binns were formed, representing the vast majority of the animal’s diet (as represented by amplicon sequencing of the feces) (Table S 2).

To visualize the composition of the various diets, we used package bipartite v2.18^85^, with taxa for which mean relative read abundance (RRA) on at least one season was ≥ 2.00%. Bipartite was also used to present diet composition by life form (woody/herbaceous) after assigning either one to each taxon. When a taxon potentially included both lifeforms, it was noted as unclassified. We used nonmetric multidimensional scaling (NMDS) ordinations of Helinger-transformed readings to visualize the relationships among and between gazelles and cattle and between seasons at the two sites.

To test dietary differences, we used *Adonis* permutational analysis of variance (PERMANOVA) with 999 permutations of Bray–Curtis dissimilarity in the R package vegan v2.6-4^86^. This approach is particularly appropriate for small sample sizes, as the reference distribution is derived directly from our observed data rather than from theoretical distributions^85,86^. When significant differences were observed, we further compared pairwise combinations, using package pairwiseAdonis v0.4.1^89^. To estimate dietary overlap, we used Bray-Curtis similarity (BCsim) between all possible sample pairs of gazelles and cattle co-occurring at a certain site and season, based on Hellinger-transformed readings. BCsim, calculated as 1 - Bray–Curtis dissimilarity, ranges between 0 for no overlap, to 1 for complete overlap. These values, calculated with package vegan, were tested with the non-parametric Kruskal-Wallis test because they were not normally distributed. Post hoc comparisons were performed by Dunn’s tests with Bonferroni’s correction, using package FSA v0.9.3^90^. We ran parallel analyses using Jaccard’s similarity index ranging from 0 (no overlap) to 1 (complete overlap). The index is based on plant taxa occurrence data (presence/absence), rather than on proportions, which is considered a more conservative approach^89^.

Given the small sample size from YNR, we ran power analyses using R package MultNonParam^90^ to validate the results of our statistical analysis. Considering 25 Bray-Curtis values per season, three seasons, a 0.05 alpha, and a logistic distribution, the model power was 0.793.

All statistical analyses were done with R 4.2.2^91^.

## Electronic supplementary material

Below is the link to the electronic supplementary material.

Supplementary Material 1

## Data Availability

Raw sequence data was deposited into NCBI SRA database under the Bioproject accession number PRJNA1178189.
