# Supplementary material for: Dietary composition and overlap between cattle and endangered mountain gazelle (Gazella gazella)
Source: Sci Rep. 2025 Jun 6;15:20002. doi: 10.1038/s41598-025-04366-w (PMC12144209; doi:10.1038/s41598-025-04366-w)
Supplement: Supplementary file 1 — Supplementary Material 1 [file 41598_2025_4366_MOESM1_ESM.docx]

Supplementary material


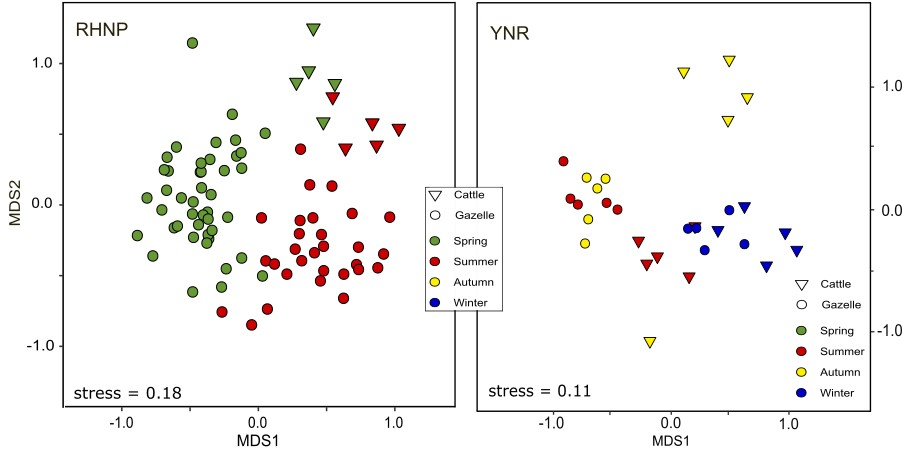


Figure S 1. Dietary Niche separation between mountain gazelles and cattle on different seasons in two study sites: Ramat Hanadiv Nature Park (RHNP) and Yehudiya Nature Reserve (YNR). NMDS ordination of Jaccard dietary dissimilarity based on presence/absence at the genus or family level. Each point corresponds to a fecal sample.


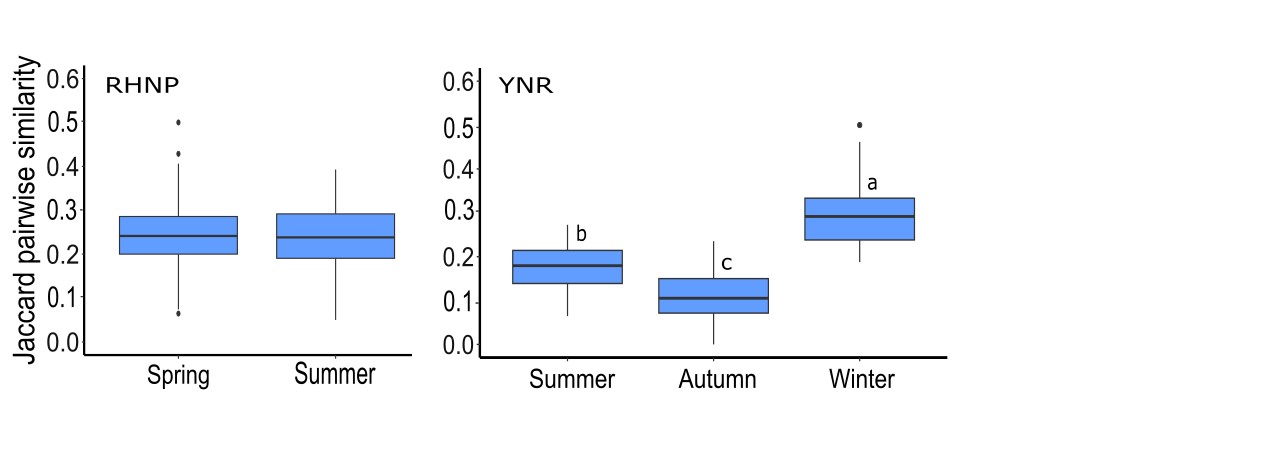


Figure S 2. The similarity between fecal samples of mountain gazelles and cattle in seasons when both ungulates co-occur - in Ramat Hanadiv Nature Park (RHNP; cattle only present during spring and summer) and in Yehudiya Nature Reserve (YNR; data for spring is missing). Values presented are Jaccard pairwise similarity between fecal samples (1 - Jaccard dissimilarity, based on presence/absence genus data). Within each site, different letters indicate statistically significant differences between seasons (*P* < 0.001), based on the Krus-kal-Wallis test and Dunn post hoc test with Bonferroni correction.


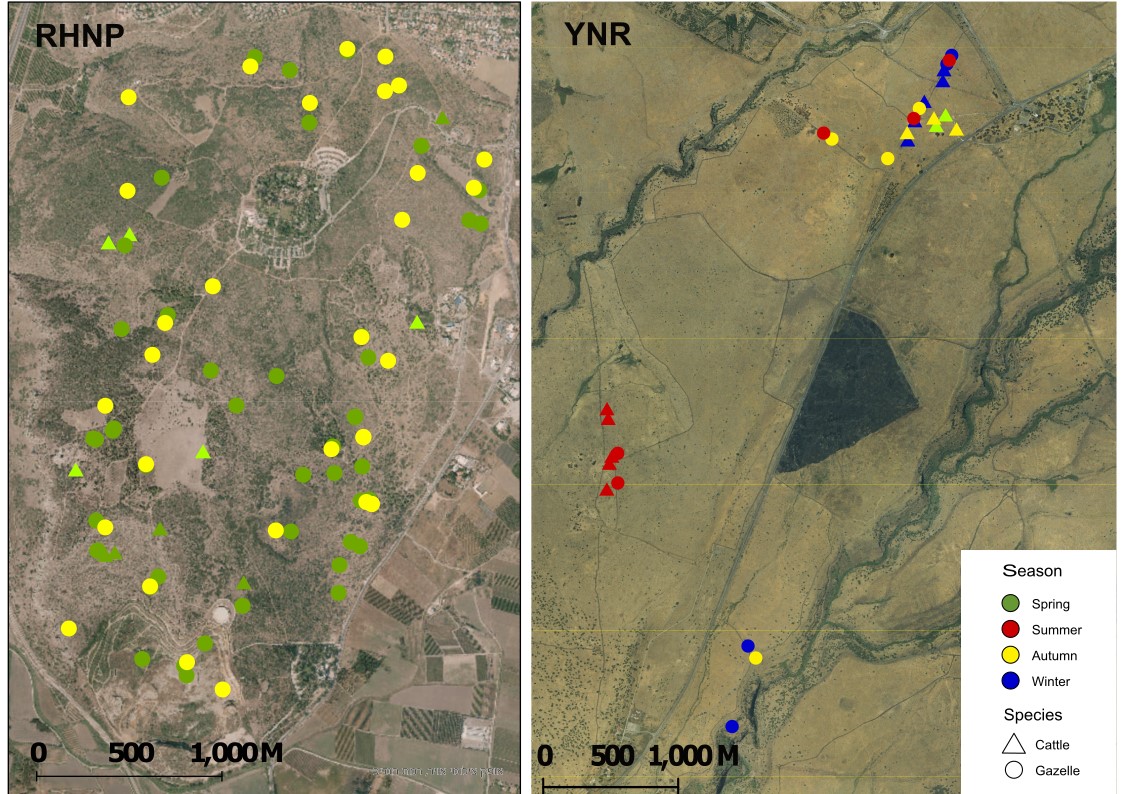


Figure S 3. The locations of mountain gazelles and cattle fecal samples, collected at two sites for DNA Metabarcoding: Ramat Hanadiv Nature Park (RHNP), and Yehudiya Nature Reserve (YNR). Maps were produced using ArcGIS Pro (version 3.03; Base Map: World Map; copyright and licensed by ESRI <https://www.esri.com/en-us/arcgis/products/arcgis-pro/overview>).

Table S 1. Permutational analyses of variance for dietary differnces between ungulate species (gazelles and cattle), season, and species × season interaction. Analyses were based on occurrence-based Jaccard dissimilarity distances, calculated for seasons when the two species co-occured – spring and summer in Ramat Hanadiv Nature Reserve (RHNP), autumn, spring and winter (spring missing) in Yahudiya Nature Reserve (YNR). P: '*' < 0.05, '**' < 0.01, '***' < 0.001.

| RHNP | | | | YNR | | | |
| --- | --- | --- | --- | --- | --- | --- | --- |
| Factor | Df | Sums Of Sqs | R^2^ | Factor | Df | Sums Of Sqs | R^2^ |
| Species *** | 1 | 1.54 | 0.05 | Species *** | 1 | 1.21 | 0.13 |
| Season *** | 1 | 2.40 | 0.07 | Season *** | 2 | 2.17 | 0.23 |
| Species × Season ** | 1 | 0.67 | 0.02 | Species × Season*** | 2 | 1.31 | 0.14 |
| Residuals | 79 | 27.93 | 0.86 | Residuals | 24 | 4.82 | 0.50 |
| Total | 82 | 32.54 | 1 | Total | 29 | 9.51 | 1.00 |

Table S 2. Number of DNA reads assigned a genus, family or order level by DNA metabarcoding, from fecal samples of mountain gazelles and cattle. In each site × season combination, the number of reads oncluded (in), or excluded (out), % of reads included out of the total reads (% in), and the number of taxa analyzed (N taxa) are given. Site are: Ramat Hanadiv Nature Park (RHNP; spring and summer), and Yehudiya Nature Reserve (YNR; summer, autumn, and winter).

| Location | Species | Season | In | Out | % in | N taxa |
| --- | --- | --- | --- | --- | --- | --- |
| RHNP | Gazelle | Spring | 279322 | 0 | 100.0 | 94 |
| RHNP | Gazelle | Summer | 415877 | 909 | 99.8 | 89 |
| RHNP | Cattle | Spring | 103334 | 299 | 99.7 | 49 |
| RHNP | Cattle | Summer | 74630 | 543 | 99.3 | 44 |
| YNR | Gazelle | Summer | 89220 | 558 | 99.4 | 43 |
| YNR | Gazelle | Autumn | 115006 | 1597 | 98.6 | 40 |
| YNR | Gazelle | Winter | 128453 | 3725 | 97.2 | 31 |
| YNR | Cattle | Summer | 77102 | 4698 | 94.3 | 33 |
| YNR | Cattle | Autumn | 101525 | 14374 | 87.6 | 36 |
| YNR | Cattle | Winter | 125133 | 11787 | 91.4 | 25 |
